# The progress of molecules and strategies for the treatment of HBV infection

**DOI:** 10.3389/fcimb.2023.1128807

**Published:** 2023-03-15

**Authors:** Youlu Pan, Heye Xia, Yanwen He, Shenxin Zeng, Zhengrong Shen, Wenhai Huang

**Affiliations:** Key Laboratory of Neuropsychiatric Drug Research of Zhejiang Province, School of Pharmacy, Hangzhou Medical College, Hangzhou, Zhejiang, China

**Keywords:** HBV, molecules, HBV life cycle, inhibitors, treatment

## Abstract

Hepatitis B virus infections have always been associated with high levels of mortality. In 2019, hepatitis B virus (HBV)-related diseases resulted in approximately 555,000 deaths globally. In view of its high lethality, the treatment of HBV infections has always presented a huge challenge. The World Health Organization (WHO) came up with ambitious targets for the elimination of hepatitis B as a major public health threat by 2030. To accomplish this goal, one of the WHO’s strategies is to develop curative treatments for HBV infections. Current treatments in a clinical setting included 1 year of pegylated interferon alpha (PEG-IFNα) and long-term nucleoside analogues (NAs). Although both treatments have demonstrated outstanding antiviral effects, it has been difficult to develop a cure for HBV. The reason for this is that covalently closed circular DNA (cccDNA), integrated HBV DNA, the high viral burden, and the impaired host immune responses all hinder the development of a cure for HBV. To overcome these problems, there are clinical trials on a number of antiviral molecules being carried out, all -showing promising results so far. In this review, we summarize the functions and mechanisms of action of various synthetic molecules, natural products, traditional Chinese herbal medicines, as clustered regularly interspaced short palindromic repeats and their associated proteins (CRISPR/Cas)-based systems, zinc finger nucleases (ZFNs), and transcription activator-like effector nucleases (TALENs), all of which could destroy the stability of the HBV life cycle. In addition, we discuss the functions of immune modulators, which can enhance or activate the host immune system, as well some representative natural products with anti-HBV effects.

## Introduction

Hepatitis B virus (HBV), a specific small hepatotropic DNA virus, is the causative agent of hepatitis B, which is now the most common serious liver infection all over the world. In 2019, the global prevalence rate of HBV reached 4.1%, representing 316 million people living with HBV. In the same year, HBV-related diseases were the causes of approximately 555,000 deaths around the world (Sheena et al., 2022). Hepatitis B is therefore regarded as a serious threat to human life and health, despite it being preventable. To solve this public health threat by 2030, the World Health Organization (WHO) put forward an ambitious target for the elimination all types of viral hepatitis, especially hepatitis B and C, in its document *Global Health Sector Strategy on Viral Hepatitis 2016–2021. Towards Ending Viral Hepatitis.*

Typically, the treatment of hepatitis B is divided into three clinical levels (Lok et al., 2017; Suk-Fong Lok, 2019). The first level is partial treatment, which results in the detection of hepatitis B surface antigens (HBsAgs), but after this finite treatment course, HBV DNA is still persistently undetectable in a patient’s serum. The second level is functional treatment, after which both HBsAg and HBV DNA can be detected in the patient’s serum, but not continuously. The third level of treatment, which is the ultimate goal, entails complete removal, resulting in HBsAg being undetectable in the patient’s serum, and in the eradication of HBV DNA (Lok et al., 2017; Suk-Fong Lok, 2019).

Traditionally, there have been mainly two strategies used in the treatment of chronic HBV infections in clinical settings, that is, antiviral drugs [i.e., nucleoside analogues (NAs)] and interferons (IFNs). In the 1990s, IFN alpha (IFN-α) was approved by the US Food and Drug Administration (FDA) for the treatment of HBV infections, which started a new era of antiviral therapy (Trepo, 2014). IFNs are endogenous cytokines produced by immune system cells in response to viral infection. Of note, pegylated IFN-α is not only injectable, and, therefore, usable in the treatment of HBV infections, but could also play a significant role in regulating host immunity (Ye and Chen, 2021). However, serious adverse reactions, including cytopenia, exacerbations of neuropsychiatric symptoms (i.e., depression and insomnia), and the production of thyroid autoantibodies associated with this treatment occurred on a frequent basis (Tang et al., 2018). As a result of the focus on the development of specific antiviral drugs, lamivudine was marketed and became the first NA approved by the FDA for the treatment of hepatitis B, meaning that a new therapy for HBV infection was available (Dienstag et al., 1999). It has been reported that NAs block the normal replication processes of HBV by inhibiting the activity of HBV polymerase (Pierra Rouviere et al., 2020). However, although NAs are effective in reducing viral load, they require long-term administration and frequently induce adverse effects, such as fatigue, dizziness, headache, and nausea (Bedre et al., 2016; Roediger et al., 2022). In addition, despite the capacities of both NAs and IFNs to significantly lower levels of viral DNA in the blood, functional cure rates after these therapies remained low (i.e., 3%–7% for pegylated IFNs and 1%–12% for NAs) (Feng et al., 2018; Tang et al., 2018). Therefore, there is an urgent need to develop more effective and ideal medicines or strategies to achieve higher functional cure rates, eventually developing a complete eradication of HBV. Hence, in this review, we summarize the existing therapeutic agents for HBV infections, including various synthetic molecules, natural products, traditional Chinese herbal medicines, clustered regularly interspaced short palindromic repeats and their associated proteins (CRISPR/Cas)-based systems, zinc finger nucleases (ZFNs), and transcription activator-like effector nucleases (TALENs), all of which have the ability to destroy the stability of the HBV life cycle. We also provide a summary of the existing anti-HBV immunotherapies and host immune modulators.

## HBV life cycle

There are some barriers to the treatment of HBV infections. The first of these is its involvement with covalently closed circular DNA (cccDNA) and integrated HBV DNA. cccDNA is the transcriptional template for DNA replication and antigen production, and, meanwhile, integrated HBV DNA can be a template for the generation of HBsAgs. In addition, a higher viral burden,associated with increasing levels of HBV DNA and HbsAG, is also a barrier to treatment. Finally, an impaired host immune response can also prevent the treatment of HBV infections (Wong et al., 2022).

**Figure 1:**
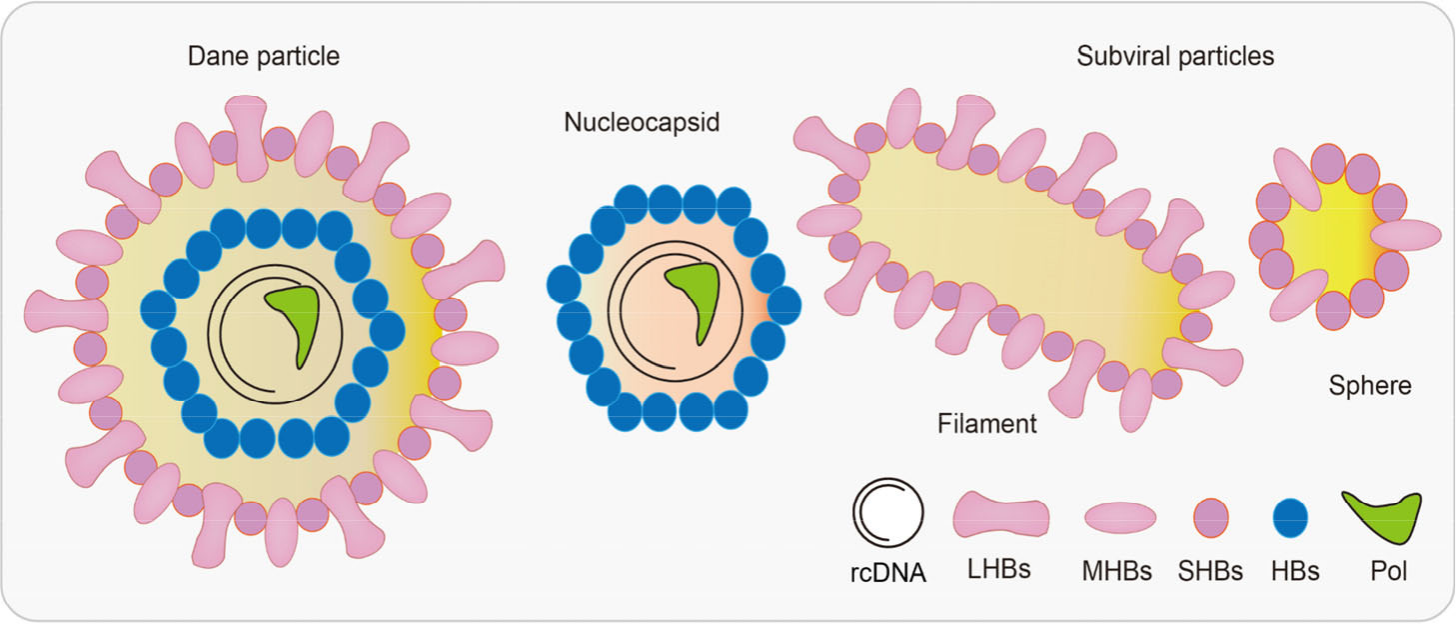
Hepatitis B virus (HBV)-associated particles. The diameter of Dane particles is about 42 nm, and their envelopes are made up of large/middle/small HBsAgs (LHBs/MHBs/SHBs). A nucleocapsid contains both viral genomes and polymerases. There are two types of subviral particles: filaments and spheres. HBV, hepatitis B virus; HBsAgs, hepatitis B surface antigens; LHBs, lipid-embedded large HBsAgs; MHBs, lipid-embedded medium HBsAgs; SHBs, lipid-embedded small HBsAgs.

Before the specifics of the HBV life cycle are detailed, it is meaningful to describe the structure of HBV. There were three common types of HBV particles observed in patients’ serum (Tsukuda and Watashi, 2020). For instance, complete HBV virions, also known as Dane particles, were in the form of spheres that were 42 nm in diameter and could routinely be detected in the blood of infected patients. In addition, the structure of Dane particles possess 3.2 kb of partially double-stranded relaxed circular DNA (rcDNA) bonded to polymerase, an inner nucleocapsid formed by the core protein hepatitis B core antigen (HBcAg), and an outer envelope generated by lipid-embedded small (SHBs), middle (MHBs), and large HBsAgs (LHBs) (**Figure 1**) (Kim et al., 2022). Comparatively, the incomplete particles, also known as the subviral particles of HBV, were of two major types, that is, the classical HBsAg spheres and filaments (**Figure 1**). In addition, there were putative particles containing HBV RNA in much lower levels than in other particles (i.e., 100- to 1,000-fold lower than in complete virions) (Roediger et al., 2022).

**Figure 2:**
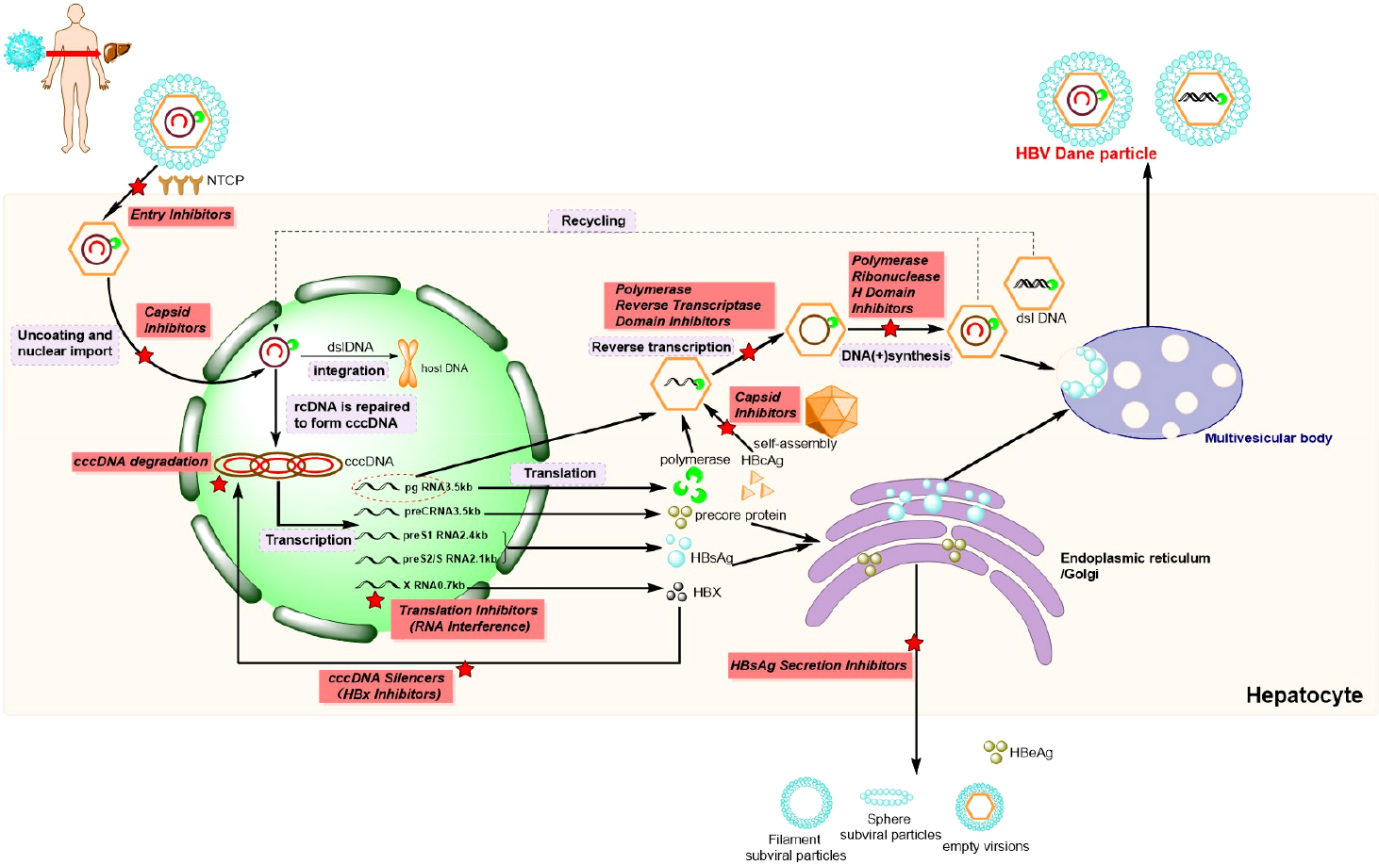
Hepatitis B virus (HBV) life cycle and potential drug targets (). HBV, hepatis B virus. NTCP, Na^+^-taurocholate co-transporting polypeptide; cccDNA, covalently closed circular DNA; rcDNA, relaxed circular DNA; dslDNA, double-stranded linear DNA; pgRNA, polyvalent guide RNA; HBcAg, hepatitis B virus core antigen; HBsAg, hepatitis B surface antigen; HBX, hepatitis B-encoded X antigen; HBeAg, hepatitis Be antigen; cRNA, cytoplasmic RNA.

Initially, HBV attaches to the host hepatocyte surface by binding to specific factors, such as heparan sulfate proteoglycans (HSPGs), in a low-affinity manner (**Figure 2**). Subsequently, the virus makes contact with the specific receptor sodium taurocholate co-transporting polypeptide (NTCP) through the pre-S1 domain of a large viral envelope protein in a high-affinity manner. Virus–receptor interactions can also trigger HBV internalization into hepatic cells in an endocytosis-dependent manner (Tsukuda and Watashi, 2020). Of note, it has been reported that the epidermal growth factor receptor (EGFR) is a host factor that interacts with NTCP and mediates HBV internalization (Iwamoto et al., 2019). After the HBV enters the cell, the viral envelope fuses with the endosome membrane and releases the free nucleocapsid into the cytoplasm. The nucleocapsid can then utilize the microtubule network to facilitate its transition into the nucleus, which occurs *via* its interaction with motor proteins (Hayes et al., 2016). In the nucleus, the rcDNA of HBV is modified and repaired into cccDNA, and a part of the incoming HBV DNA can also be integrated into the host’s genome (Tsukuda and Watashi, 2020). Significantly, cccDNA can reinitiate infection after long-term antiviral therapy, which is typically the main reason for HBV infections not being cured completely.

The cccDNA in the nucleus functions as a template for the transcription of different lengths of messenger RNAs (mRNAs), which can thereafter be released into the cytoplasm to enable the translation of the corresponding proteins, including polymerases, HBcAgs, precore proteins, HBsAgs, and hepatitis B virus X proteins (HBxs) (Wang et al., 2020). Synthesized HBcAg monomers are initially combine to yield a dimer, which can subsequently constitute an icosahedral capsid by self-assembly. Thereafter, partial polyvalent guide RNAs (pgRNAs), along with polymerases, are encapsulated into icosahedral capsid to generate the core proteins (Tsukuda and Watashi, 2020). Subsequently, after the catalysis of the reverse transcriptase region of the HBV polymerase and HBV ribonuclease H (RNaseH) in the nucleocapsid, pgRNA serves as the template for the strand DNA. After post-translational modifications within the endoplasmic reticulum and Golgi apparatus, the mature viral particles are secreted out of the infected hepatocyte, and this occurs contemporaneously with the secretion of a large number of non-infectious particles. Finally, the mature hepatitis Be antigen (HBeAg), generated from precore polypeptides, can be released directly into circulation (Wang et al., 2020), so as to regulate the immune response to the intracellular nucleocapsid. As shown in **Figure 2**, every step of the HBV life cycle can be catalyzed by corresponding enzymes, and, therefore, serves as a potential drug target for the development of novel anti-HBV medicines or theoretical methods of treatment.

## The development of drugs affecting the HBV life cycle

At present, there have already been drugs and active compounds developed for the treatment of HBV infection. The following mainly introduced the structures and relevant properties, including small molecules, RNAs, peptides, and NPs. The following text introduces their structures and describes the relevant properties of these molecules.

### Entry inhibitors

It had been reported by Yan’s group in 2012 that the NTCP could be an acceptable drug target for the treatment of HBV infection (Yan et al., 2012). Myrcludex B (**Table 1**), a first-in-class entry inhibitor, is a chemically synthesized polypeptide consisting of 47 amino acids, and it also has the preS1 domain of HBV large surface proteins (Uhl et al., 2016; Chen et al., 2022). Myrcludex B competes with HBV for NTCP receptor sites to prevent it from entering hepatocytes. This drug was approved in 2020 by the European Union (EU) for the treatment of chronic hepatitis D virus (HDV) and of HBV infections in phase 1/11 trials (Kang and Syed, 2020). The findings of a phase I trial indicated that treatment with Myrcludex B was well tolerated by participants, with no serious or relevant adverse reactions occurring (Cheng et al., 2021a). In addition, it also demonstrated a high potential foreffective, combined use with IFNs and NAs.

**Table 1:** Summary of molecules as entry inhibitors.

Structure	Target	Company	NCT number	Clinical status
**GTNLSVPNPLGFFPDHQLDP AFGANSNNPDWDFNPNKDHWPEANKVG** **Myrcludex B**	NTCP	Gilead Sciences Inc.	NCT02881008 NCT04166266 NCT02637999	Approved by the EU for the treatment of chronic HDV; phase I/II trial(s)
**Undisclosed** **HH-003**	NTCP	Huahui Health Ltd	NCT05542979	Phase I trial(s) in Australia; phase II trial(s) in China
**Undisclosed** **HH-006**	NTCP	Huahui Health Ltd	NCT05275465	Phase I trial(s)
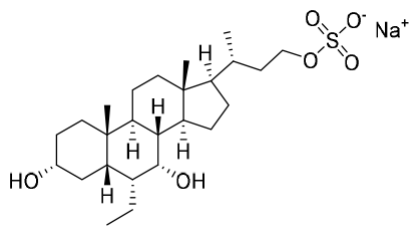 **INT-767**	FXR	Intercept Pharma	Undisclosed	Phase I trial(s)
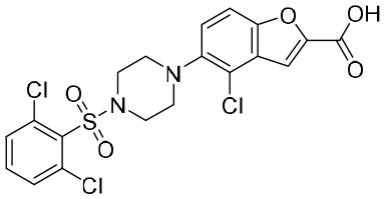 **Vonafexor**	FXR	ENYO Pharma	NCT03469583 NCT03320616 NCT04365933 NCT03272009	Phase II trial(s)

EU, European Union; FXR, farnesoid X receptor; HDV, hepatitis D virus; NCT, National Clinical Trial; NTCP, sodium taurocholate co-transporting polypeptide.

Monoclonal antibodies were also efficient as HBV entry inhibitors (Wi et al., 2017). HH-003 and HH-006 (**Table 1**), full human monoclonal antibodies developed by Huahui Health Ltd, targeted the preS1 domain of the HBV large surface protein (Yang and Xie, 2022). These two antibodies could suppress the binding of HBV with NTCP receptor sites to block HBV infection. To date, phase II trials of HH-003 injection have been conducted in China, and a phase I trial of HH-006 injection is currently under way in Australia.

It has also been reported that, under normal conditions, farnesoid X receptor (FXR) can down-regulate the expression of NTCP *via* the induction of small heterodimer partner (SHP) nuclear receptors (Farooqui et al., 2022). INT-767, a bile acid (BA) derivative, was also identified as a specific FXR agonist and could effectively block the entry of HBV by down-regulating the expression of NTCP (Ito et al., 2021). In chimeric mice with humanized liver, INT-767 markedly delayed the initial increase in HBsAg, hepatitis B e antigen (HBeAg), and HBV DNA levels, as well as reducing levels of cccDNA (Ito et al., 2021). After a series evaluation of pharmacokinetic (PK) and pharmacodynamic (PD) properties, INT-767 became a candidate for the treatment of HBV infections and a phase I study evaluating its possible use as a treatment for HBV was conducted (Ito et al., 2021). In addition, INT-767 was also evaluated for the treatment of non-alcoholic steatohepatitis (NASH) and intestinal ischemia reperfusion injury (IRI) because of its function as a dual FXR/Takeda G protein-coupled receptor 5 (TGR5) agonist (Anfuso et al., 2020).

A clinical study primarily evaluating the safety and antiviral effect of vonafexor, another FXR agonist with a benzofuran-2-carboxylic acid moiety, was also conducted (Erken et al., 2021). The corresponding experimental results, as well as those of a phase II trial, demonstrated that vonafexor was safe, and that its use resulted in an observable decline in the number of HBV markers observed in chronic hepatitis B (CHB) patients (Erken et al., 2021). In addition, as with INT-767, the presence of vonafexor was also identified in a phase IIa assessment of non-alcohol steatohepatitis (NASH) (Fiorucci et al., 2020).

In addition to the drugs discussed above, several marketed drugs have also been identified as efficient inhibitors of the NTCP–LHB interaction, such as the immunosuppressive agent cyclosporine A and its derivatives (Nkongolo et al., 2014), angiotensin II receptor antagonist irbesartan (Ko et al., 2015), and anti-hyperlipidemia agent ezetimibe (Lucifora et al., 2013).

### Inhibition of assembly or formation of the cccDNA minichromosome

Because of the key role HBV cccDNA plays in viral persistence, the removal, destruction, or inhibition of cccDNA are regarded as the keys to virus eradication. In the nucleus, HBV cccDNA is formed from rcDNA, which binds with both histones (i.e., H2A, H2B, H3, H4, and H1) and non-histone proteins (i.e., HBcs, HBxs, and host factors), and is then organized into a chromatin-like structure termed as the HBV cccDNA minichromosome (Zhang et al., 2021). Because of the Histone Acetyltransferase 1 (HAT1) regulating the HBV cccDNA minichromosome, it had been a promising target for controlling the assembly of the cccDNA (Yang et al., 2019). Nevertheless, to date, relative inhibitors have not been reported.

Notably, two disubstituted sulfonamides (DSS), namely CCC-0975 and CCC-0346 (**Table 2**), have been identified as the novel inhibitors of HBV cccDNA biosynthesis through a specific screening approach (Cai et al., 2012). These two compounds could interfere with the conversion of rcDNA to cccDNA, and further research into their function might ultimately change the landscape of hepatitis B treatment.

**Table 2:** A summary of compounds that interfere with the function of cccDNA minichromosomes.

Structure	Mechanism of action	Activity	PK/PD	Clinical status
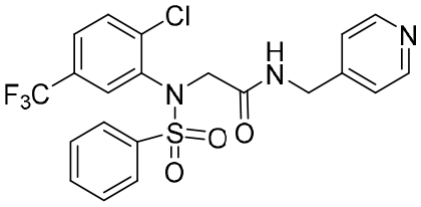 **CC-0975**	Interferes with cccDNA synthesis	HepDE19 cells: EC_50_ = 4.55 μM; CC_50_ > 50 μM; CC_50_/EC_50_ > 11	Undisclosed	Preclinical
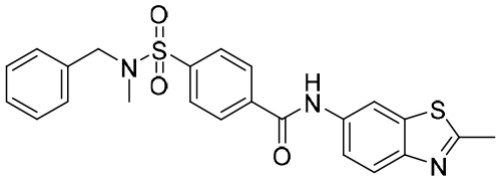 **CC-0346**	Interferes with cccDNA synthesis	HepDE19 cells: EC_50_ = 0.35 μM; CC_50_ = 2.57 μM; CC_50_/EC_50_ = 7.34	Undisclosed	Preclinical
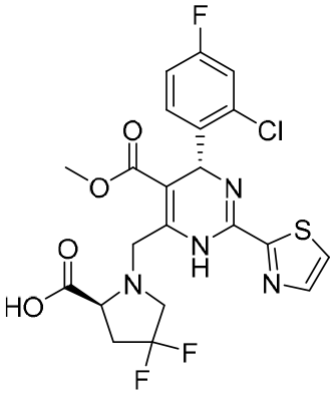 **HAP_R01**	Binds Cp149	HepG.2.2.15 cells: EC_50_ = 0.003 μM; CC_50_ = 65 μM; CC_50_/EC_5_ = 21,667	Undisclosed	Preclinical
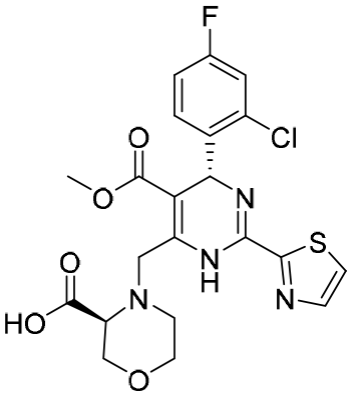 **HAP_R10**	BindsCp149	HepG.2.2.15 cells: EC_50_ = 0.003 μM; CC_50_ > 100 μM; CC_50_/EC_50_ > 33,333	Mice plasma: CL (iv): 19 mL/min/kg; *t*_1/2_ 1.5 h; *F* (%): 37; *C*_max_: 413 μg/L; AUC (PO): 962 μg/L/h	Preclinical

Clearance (CL), Area Under Curve (AUC), Per Os (PO), Pharmacokinetics (PK), Pharmacodynamics (PD), concentration for 50% of maximal effect (EC50), 50% cytotoxicity concentrations (CC50), t1/2, half-life; Cmax, maximum concentration; Tmax, time to peak.

**Figure 3:**
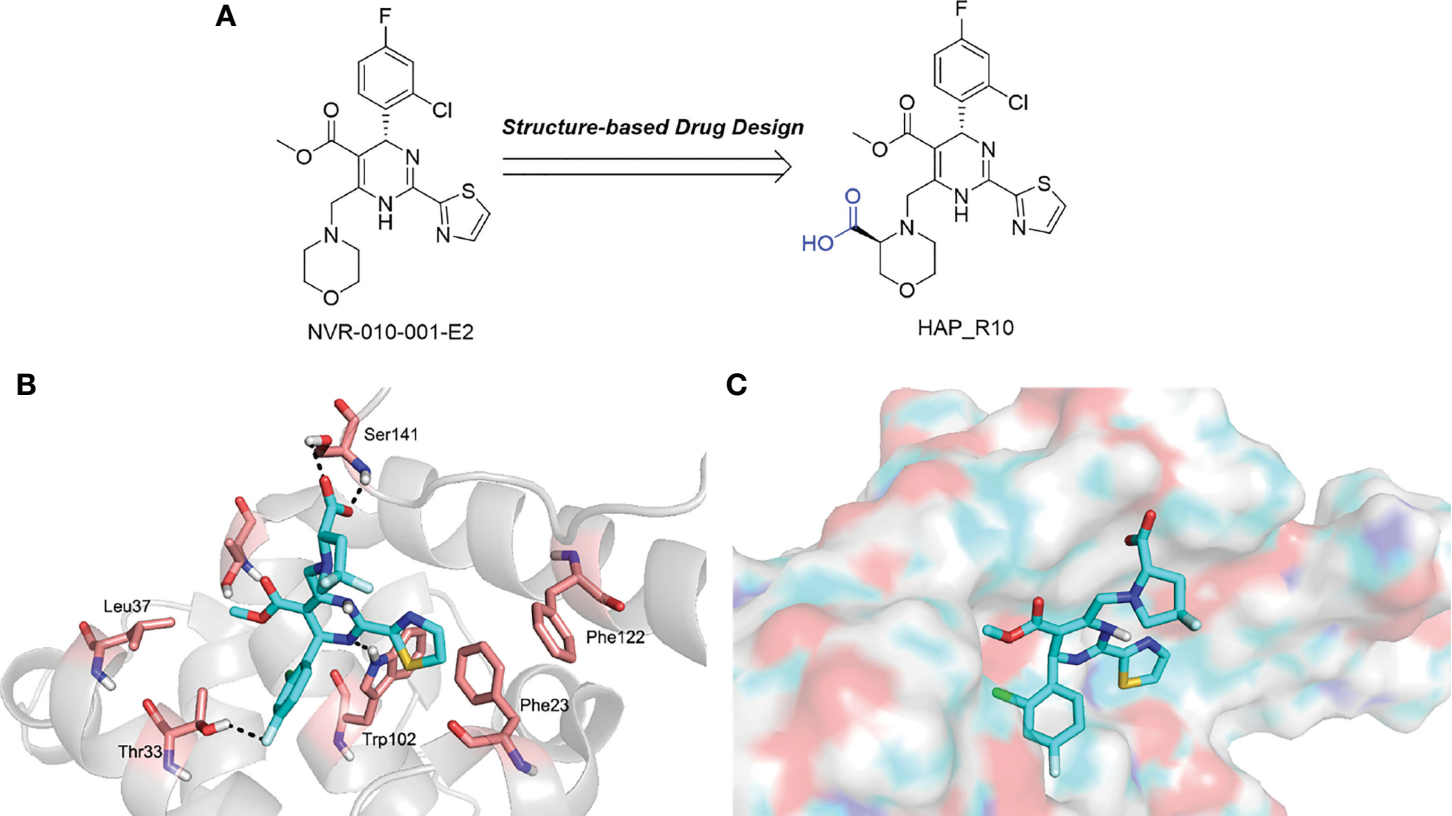
**(A)** The design of HAP_R10; **(B)** The interactions of HAP_R01 with Cp149; **(C)** The binding mode of HAP_R01. Cp149, the 149-residue core protein assembly domain.

In addition to the molecules and compounds discussed above, HBV core protein allosteric modulators (CpAMs) could also efficiently inhibit HBV reproduction by modulating the assembly of capsids at a late stage (Mak et al., 2017). It has also been reported that HAP_R01 and HAP_R10, which are 4−H heteroaryldihydropyrimidine (HAP) analogues, can alter capsid integrity to affect HBV infectivity and suppress cccDNA formation by inhibiting the activity of C-terminally truncated proteins (i.e., Cp149) (Qiu et al., 2017). As a third-generation 4-H HAP inhibitor, HAP_R10 is optimized from NVR-010–001-E2 by the introduction of carboxyl groups (**Figure 3A**), and subsequently selected for further development as an oral anti-HBV agent (Qiu et al., 2017).

Importantly, the co-crystal structure of HAP_R01 (**Table 2**) with Cp149 has been determined (PDB ID: 5WRE) (Zhou et al., 2017). As shown in **Figure 3B**, in addition to the H-bond interaction of the parent nucleus with Thr33 and Trp102, the carboxyl group can also generate a H-bond interaction with the Ser141 side chain. In addition, thiazole moiety can also be inserted into a specific hydrophobic pocket that is surrounded by Phe23 and Phe122, thereby forming a strong hydrophobic interaction (**Figure 3C**). Because of these crucial interactions, HAP_R01 became a potent inhibitor and guided the further structure optimization.

### Silencing cccDNA transcription

Accumulating evidence has proved that epigenetic modifications of cccDNA contribute to viral replication and chronic HBV infection (Hong et al., 2017). With the goal of silencing cccDNA in infected hepatocytes, epigenetic therapy might be a promising therapeutic strategy.

**Figure 4:**
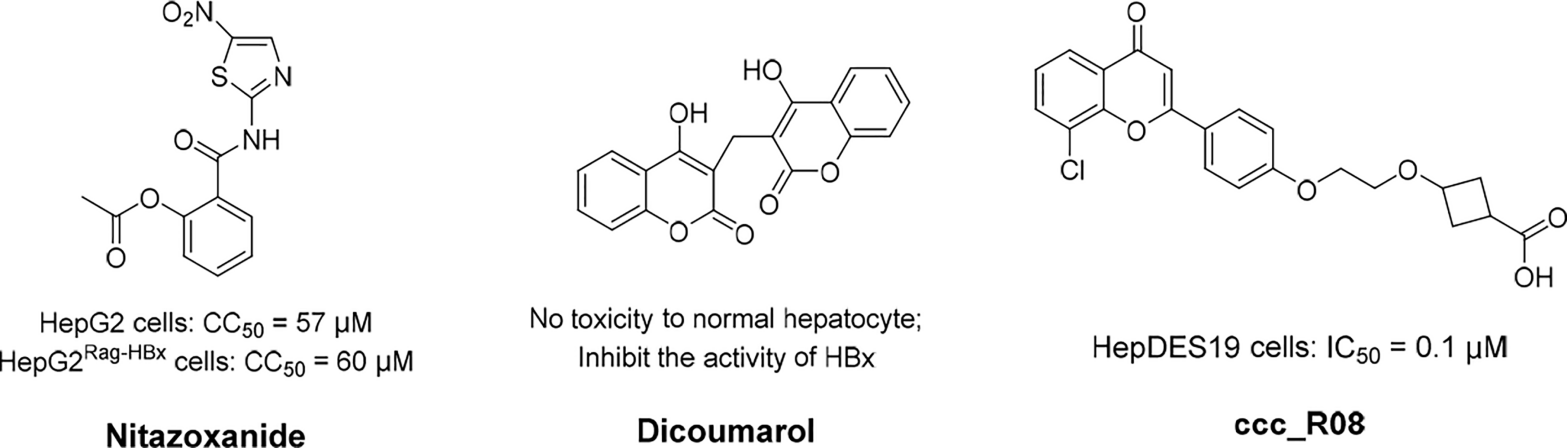
The structures of nitazoxanide, dicoumarol, and ccc_R08. Concentration for 50% of maximal effect (EC50), 50% cytotoxicity concentrations (CC50), the half maximal inhibitory concentration (IC50).

A significant curative strategy, that is the functional silencing of cccDNA, might be achieved by targeting the viral protein HBx because of its crucial role in stimulating the transcription of HBV cccDNA (Sekiba et al., 2022). Furthermore, HBx can enable cccDNA transcription by means of inactivating the cellular damage-specific DNA-binding protein 1 (DDB1). Notably, DDB1 contains E3 ubiquitin ligase and could degrade structural maintenance of chromosomes 5/6 (SMC5/6), which bonds with the cccDNA minichromosome (Sekiba et al., 2022). In addition, HBx prevents transcriptional repressor recruitment to the cccDNA minichromosome (Zhang et al., 2021). In brief, HBx can activate the transcription of host genes by directly interacting with nuclear transcription factors, or activating various signal transduction pathways in the cytoplasm (Hong et al., 2017). Nitazoxanide (NTZ, **Figure 4**), a thiazolide anti-infective agent, has been approved by the FDA for the treatment of protozoan enteritis (Sekiba et al., 2019). Interestingly, it has been discovered that NTZ efficiently inhibits the protein interaction of HBx with DDB1, which in turn leads to a significant reduction in viral transcription activity, and, therefore, the number of viral products (Sekiba et al., 2019). Recently, much more indepth research of NTZ for the treatment of HBV infection has been going on. In addition, it has been proven that an NQO1 inhibitor, dicoumarol (**Figure 4**), can exerts an anti-HBV influence, in that it promotes the degradation of HBx and blocks cccDNA transcription (Cheng et al., 2021b). Further experimental results demonstrate that dicoumarol is capable of potent antiviral activity that restricts the production of HBV RNAs, HBV DNA, HBsAgs, and HBc proteins in HBV-infected cells and in a humanized-liver mouse model.

### Destroying the stability of cccDNA

In the destabilization process for HBV cccDNA, a number of tools for editing genomes were reported, such as CRISPR/Cas 9 mechanism systems, ZFNs, and TALENs. All of these representative gene-editing approaches can cause a nick in double-stranded DNA at a specific target region in turn changing the specific DNA sequence in said region (Bhat and Kazim, 2022).

The CRISPR/Cas9 mechanism technique is the most famous, and has garnered considerable interest because of its accessibility and versatility. By designing the guide RNA (gRNA), so that it is complementary to the target DNA sequence, the CRISPR/Cas9 mechanism can be redirected to specifically cleave any desired DNA genome, resulting in site-specific DNA double-strand breaks (DSBs). Although the CRISPR/Cas9 mechanism technique represents a promising therapeutic approach, there are some challenges that must be overcome before it can be applied in a clinical setting. For instance, the cleavage of integrated HBV DNA by the CRISPR/Cas9 mechanism might cause DSBs to occur in the host genome, in turn giving rise to serious safety concerns related to host genome instability and carcinogenesis. In addition, the off-target effects, the difficulty in finding conserved target of HBV sequences, and *in vivo* delivery efficiency also hinder the extensive application of the CRISPR/Cas9 mechanism (Komor et al., 2016). However, excitingly, research indicates that newly developed CRISPR-derived base editors (BEs) could permanently inactivate the HBV genome by introducing irreversible point mutations for the formation of premature stop codons, without affecting the host genome (Yang and Yang, 2022). The application of Cas9 with high fidelity and the broad protospacer adjacent motif (PAM) in the treatment of hepatitis B could also further reduce the impact [or severity] of off-target effects and increase the capacity of gRNA pools to target conserved HBV DNA sequences across different genotypes of HBV (Kleinstiver et al., 2015). As for the delivery efficiency by lipid nanoparticles *in vivo*, the non-viral delivery of Cas9 mRNA and ribonucleoproteins has considerable potential for use in liver-targeted delivery in clinics (Gillmore et al., 2021). In view of the research progress detailed above, CRISPR/Cas9 therapy might yet provide the ultimate approach to developing a cure for HBV.

Zinc-finger nucleases (ZFNs) could introduce a DSB into a desired target and significantly upregulate gene targets by activating cellular DNA repair pathways (Zakirova et al., 2020). Weber’s group developed ZFNs targeting HBV polymerase, including X and core genes, and delivered them into HepAD38 cells by self-complementary adeno-associated virus vectors. Surprisingly, HBV-ZFNs could efficiently induce site-specific mutations, and the activity of these HBV-targeted ZFNs resulted in the sustained suppression of HBV DNA replication. Notably, high specificity was observed for all ZFNs, and their off-target effects were negligible (Weber et al., 2014).

Moreover, it has been shown that TALENs also possess properties similar to ZFNs (Bhardwaj and Nain, 2021). Bloom’s group engineered mutagenic TALENs that targeted four HBV-specific sites within the viral genome (Dreyer et al., 2016). The researchers discovered that TALENs with cognate sequences in the S or C open-reading frames (ORFs) efficiently disrupted HBV sequences at the intended sites and suppressed the production of markers related with viral replication. To maximize this durable antiviral effect, Dreyer’s group put forward a strategy that combined gene editing with homology-directed DNA recombination (HDR), to introduce HBV silencing artificial primary microRNAs (miRNAs) into HBV DNA targets (Bloom et al., 2013).

Epigenetic modification could alter the status of transcribed DNA to transcriptionally inactive without changing the nucleotide sequence. Thereafter, DNA-binding domains can predefine cccDNA sequences for targeted modifications. There are two major forms of HBV epigenetic regulation: the posttranslational modification of histone proteins associated with the cccDNA mini-chromosome, and the DNA methylation of viral and host genomes (Hong et al., 2017). Correspondingly, HBV DNA modifiers mainly comprised histone acetyltransferases/deacetylases (HATs/HDACs) (Belloni et al., 2009), lysine methyltransferases (Hayashi et al., 2016), protein arginine methyltransferases (Zhang et al., 2017b), and DNA methyltransferases (DNMTs) (Zhao et al., 2017), acting in cooperation with viral factors such as HBxs and HBcAgs. As a novel first-in-class molecule cccDNA destabilizer, ccc_R08 (**Figure 4**) can target pre-existing viral genome reservoirs and displayed a robust and sustained suppression of HBsAg, HBeAg, HBV DNA, and HBV RNA levels in patient serum (Wang et al., 2022). In addition, the reduction of cccDNA levels in the liver of an experimental mouse model was also detected. Recently, the workings of this molecule were investigated in a preclinical study.

Although gene editing technology alongside the use of small-molecule drugs could destroy cccDNA, there have been many (and there are likely still unknown) challenges associated with the implementation of these therapy methods. Hence, the goal of developing a complete cure using gene editing therapy, by which all HBV genomes can be purged, will likely be achieved only at the end of the long journey to eradicate hepatitis B.

### RNA interference

After the outbreak of the coronavirus disease in 2019 (COVID-19), the mRNA vaccines have driven the research and development of nucleic acid drugs and these types of vaccines became one of the hottest research fields in the world. In comparison to traditional small-molecule drugs and antibodies, the biggest advantage of nucleic acid drugs was that they have a notably shorter development timeline (Kulkarni et al., 2021). For instance, different nucleic acid drugs could be swiftly developed by changing the specific DNA sequence. Recently, the development of small nucleic acid drugs has exponentially increased (**Table 3**). In general, small nucleic acid drugs possess 12–30 single or double strands of nucleotides, including allele-specific oligonucleotides (ASOs), small-interfering RNAS (siRNAS), and miRNAs (Kulkarni et al., 2021).

**Table 3:** Summary of molecules for RNA interference.

Name	Type	Company	Target(s)	NCT number	Clinical status
**ARC-520**	siRNA	Arrowhead Pharmaceuticals	Undisclosed	NCT02349126 NCT01872065 NCT01872065	Terminated during phase II trial(s)
**ARB-1467**	siRNA	Arbutus Biopharma Corp.	HBV S ORF; HBV X ORF	NCT02631096	Phase II trial(s) complete
**AB-729**	siRNA	Antios Therapeutics Inc.	HBV X protein	NCT04980482 NCT04820686 NCT04847440	Phase II trial(s)
**RG-6346**	siRNA	F. Hoffmann-La Roche AG	HBV S ORF	Undisclosed	Phase I trial(s)
**JNJ-3989**	siRNA	Integrity Bio Inc.	HBV X and HBV S domains	NCT04667104 NCT05275023 NCT04439539	Phase II trial(s)
**ALG-125755**	siRNA	Aligos Therapeutics Inc.	Undisclosed	NCT05561530	Phase I trial(s)
**RO7062931**	ASO	Roche	Conserved sequence in the shared 3′-region	NCT03505190 NCT03038113	Phase I trial(s)
**GSK3389404**	ASO	GSK	HBV X ORF	NCT03020745 NCT02647281	Phase I trial(s)
**Bepirovirsen**	ASO	GSK	Undisclosed	NCT05276297 NCT05630807 NCT05630820	Phase III trial(s)
**ALG-020572**	ASO	Aligos Therapeutics Inc.	HBV S ORF	NCT05001022	Phase I trial(s)

ASO, allele-specific oligonucleotide; HBV S, hepatitis B virus S; HBV X, hepatitis B virus X; HBV S ORF, hepatitis B virus S open-reading frame; HBV X ORF, hepatitis B virus X open-reading frame; NCT, National Clinical Trial.

There are already a series of siRNAs and ASOs to be used for the treatment of HBV infections in development (Hui et al., 2022). Initially, Wooddell et al. reported the efficacy of siRNA in suppressing HBV in animal models (Wooddell et al., 2013). As the first siRNA for CHB, ARC-520 could induce a reduction in HBsAg levels of 3.0 log_10_ IU/mL and 2.7 log_10_ IU/mL in mice and chimpanzees, respectively (Wooddell et al., 2013; Wooddell et al., 2017). However, because of the mortality induced by the excipient, the development of ARC-520 was terminated (Hui et al., 2022).

A phase II trial completed in 2018 demonstrated that ARB-1467 can target the ORFs of HBV S and HBV Xdomains (Hui et al., 2022). Moreover, it was shown that AB-729 and RG-6346, subcutaneous *N*-acetylgalactosamine (GalNAc)-conjugated siRNAs, can target the HBV X and HBV S domains, respectively. Comparatively, JNJ-3989 could target both the HBV X and HBV S domains with subcutaneous injection (Hui et al., 2022). Excitingly, AB-729, RG-6346, and JNJ-3989 showed good efficacy in a clinical study. Moreover, it has been reported that AB-729 and RG-6346 could play a significant role in inducing immune reconstitution against HBV (Hui et al., 2022). In addition, ALG-125918 and ALG-125755, two siRNAs, were tested in a preclinical study. Importantly, ALG-125918 was designed with a novel 5′-cap phosphate mimic to enhance siRNA loading and cleavage efficiency.

In addition to siRNAs, four ASO agents, including RO7062931, GSK3389404, bepirovirsen and ALG-020572, were also in a clinical study. RO7062931 is a subcutaneous GalNAc-conjugated ASO targeting a highly conserved sequence in the shared 3′-region. Similarly, GSK3389404 was also a GalNAc-conjugated ASO with subcutaneous injection, and could target the HBV X ORF (Hui et al., 2022). Bepirovirsen (GSK3228836) is the unconjugated version of GSK3389404, and 300 mg of bepirovirsen, administered by injection could reduce HBsAg levels to 1.99 log_10_ IU/mL (Yuen et al., 2021). ALG-020572, targeted the HBV S ORF, was absorbed and distributed rapidly in mice and displayed an intrahepatic half-life of 12 days in non-human primates. At present, ALG-020572 is being evaluated in phase I.

### Capsid assembly modulators

Hepatitis B virus (HBV) capsids have numerous functions in the HBV life cycle, such as providing sites for reverse transcription, packaging genomes, and facilitating intracellular transport (Kuduk et al., 2022). Therefore, HBV capsids could be an appropriate target for the development of active compounds. Capsid assembly modulators (CAMs) can mainly suppress HBV replication by interfering with HBV capsid assembly and the encapsidation of pgRNA. In addition, it has been found that CAMs can inhibit the establishment and replenishment of cccDNAs by interfering with capsid disassembly and the intracellular recycling of HBV nucleocapsids (Wong et al., 2022). Because of the significant role core proteins play in the HBV nucleocapsid, CAMs can also be referred to as core protein allosteric modulators (CpAMs) and promising antiviral strategy to eradicate HBV (**Table 4**). In addition, CAMs can be inserted into a specific hydrophobic pocket (i.e., the HAP pocket), located at the interface between core protein dimers, to disrupt capsid assembly and packaging of the HBV pgRNA and DNA polymerases (Bourne et al., 2006; Zhang et al., 2019). CAMs can be categorized into two types: type I CAMs (CAMI) and type II CAMs (CAMII). Of note is that it has been reported that CAMII can promote nucleation and the formation of empty capsids without packaging HBV pgRNA and DNA polymerases (Zhang et al., 2019).

**Table 4:** Summary of CAMs.

Structure	Company	Activity	PK/PD	NCT number	Clinical status
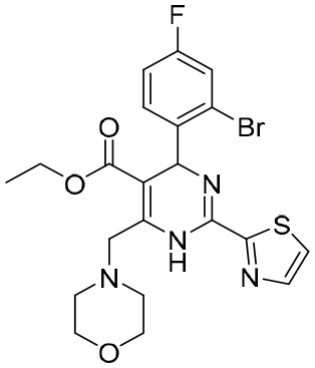 **GLS4**	Sunshine Lake Pharma Co., Ltd	HepG2.2.15 cells: EC_50_: 1 nM Lamivudine-resistant and entecavir-resistant HBV strains with EC_50_ values of 10 to 20 nM	24 h: *C*_max_ (ng/mL): 885 *t*_1/2_: 13.1 h AUC_0–24_ (ng × h/mL): 4,268	NCT04147208 NCT03638076	Phase I/II trial(s)
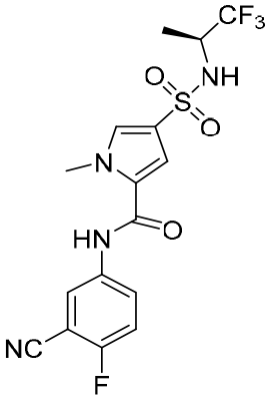 **JNJ-56136379**	Janssen Research & Development Inc.	75-mg Asian group: HBV-DNA Mean (SD) baseline (log_10_ IU/mL): 6.90 (1.91) HBV-RNA mean (SD) baseline (log_10_-p/mL): 5.59 (2.37)	75 mg Asian group: *C*_max_ (ng/mL): 1,832 *T*_max_: 12 h AUC (ng × h/mL): 36,202	NCT03361956 NCT03982186 NCT04129554	Phase I/II trial(s)
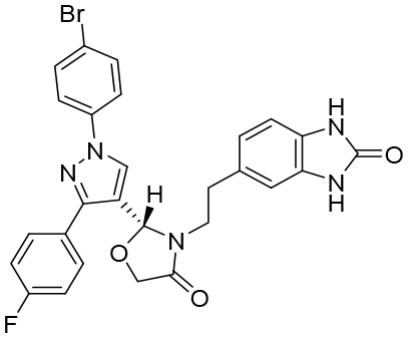 **ZM-H1505R**	Shanghai Zhimeng Biopharma, Inc.	EC_50_: 12 nM	*T*_max_: 2.5–3.5 h *t*_1/2_: 11.98–13.00 h	NCT05484466	Phase II trial(s)
Undisclosed **QL-007**	Qilu Pharmaceutical Co., Ltd	Undisclosed	Undisclosed	NCT04157699NCT04157257	Phase II trial(s)
Undisclosed **EDP-514**	Enanta Pharmaceuticals Inc.	HepAD38 EC_50_: 18 nM HepDE19 EC_50_: 27 nM HepG2.2.15 EC_50_: 17 nM	*T*_max_: 2.0–3.5 h *t*_1/2_: 15.5–26.1 h	NCT04008004 NCT04470388 NCT04783753	Phase I trial(s)
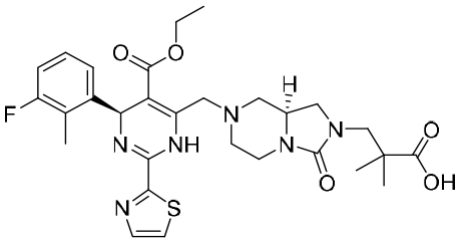 **RG-7907**	Roche	HepG2.2.15 cells: EC_50_: 6.1 ± 0.9 nM CC_50_: > 100 μM	Rapid absorbed; there was no accumulation after 28 days’ dosing	NCT02952924	Phase I trial(s)
Undisclosed **ABI-H3733**	Assembly Biosciences Inc.	HepAD38 cells: EC_50_: 5 nM PHH cells: EC_50_: 12 nM	*t*_1/2_: 18.4–23.8 h *C*_max_ (ng/mL): 2,121–4,156 AUC_0-last_ (h × ng/mL): 46,380–128,600	NCT04271592 NCT05414981	Phase I trial(s)
Undisclosed **ALG-000184**	Aligos Therapeutics Inc.	EC_50_: 1.98 nM	*t*_1/2_: 6.9–8.0 h *T*_max_: 1–3.5 h	NCT04536337	Phase I/II trial(s)
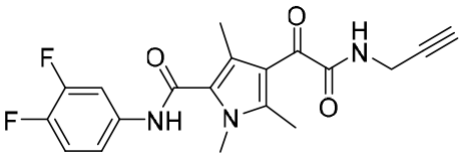 **GLP-26**	Emory University School of Medicine	EC_50_: 3 nM	*t*_1/2_: 0.77 h *T*_max_: 0.40 h *C*_max_ (ng/mL): 2,513	Not available	Preclinical trial(s)
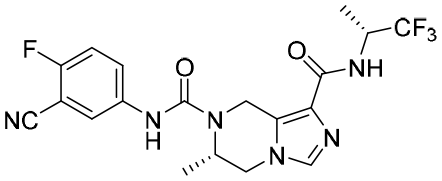 **SHR-5133**	Shanghai Hengrui Pharmaceutical Co., Ltd	EC_50_ = 26.0 nM; CC_50_: > 10,000 nM.	CL (mL/min/kg): 5.3 (mouse), 4.5 (rat), 1.1 (dog); *in vivo* efficacy: 2.32 log-HBV DNA reduction @ 30 mpk	Not available	Preclinical trial(s)

CAMs, capsid assembly modulators; NCT, National Clinical Trial. Clearance (CL), Area Under Curve (AUC), Per Os (PO), Pharmacokinetics (PK), Pharmacodynamics (PD), concentration for 50% of maximal effect (EC50), 50% cytotoxicity concentrations (CC50), t1/2: half-life; Cmax, maximum concentration; Tmax, time to peak.

It has been found that GLS4 (Morphothiadine), a heteroaryl dihydropyrimidine compound from Bay 41-4109, is the first HBV capsid assembly modulator (CAMI) that can inhibit HBV replication (Brezillon et al., 2011). It has been reported that GLS4 can disrupt or misdirect the assembly of the HBV capsid; however, at high doses, it has also been found to be hepatotoxic in rats (Brezillon et al., 2011). GLS4 demonstrated potent inhibitory activity in HBV HepG2.2.15 cells (EC_50_ 1 nM), and exhibited high potency against various mutated HBV polymerases, such as lamivudine and entecavir-resistant HBV strains with EC_50_ values of 10 to 20 nM (Ren et al., 2017). A preclinical trial indicated that GLS4 was well tolerated, and is not associated with serious adverse events or dose-limited toxicity. However, trial results also showed that the required concentration for effective antiviral activity could not be reached using GLS4 alone (Zhao et al., 2019). At present, a phase II trial of a novel therapeutic regimen, in which GLS4 and small doses of ritonavir are used to enhance plasma concentrations, is underway.

A phase II trial on JNJ-56136379 (bersacapavir), a novel and potent CAMII modulator, found that it could accelerate both the rate and extent of HBV capsid assembly *in vitro* (Berke et al., 2020). It can also suppress pgRNA encapsidation and the regeneration of cccDNA by interfering with capsid disassembly (Gane et al., 2022). JNJ-56136379 was well tolerated and demonstrated dose-dependent PK properties. In more than half of patients administered with JNJ-56136379, an obvious reduction of the HBV DNA and RNA levels was observed (Zoulim et al., 2020). However, JNJ-56136379 did not have an effect on the levels of HBsAgs and HBeAgs, and viral rebound was observed after the end of treatment (Gane et al., 2022; Taverniti et al., 2022).

A phase II trial evaluating the effectiveness of ZM-H1505R, a small-molecule HBV capsid assembly modulator with a novel pyrazole moiety for the treatment of CHB, was also carried out (Jiang et al, 2023). It was reported that ZM-H1505R was safe and well tolerated, and its plasma exposure was above its effective inhibitory concentration. In addition to this, a phase II trial evaluating the effectiveness of both ZM-H1505R and QL-007 in the treatment of CHB was carried out (Jiang et al, 2023).

Currently, drug development for anti-HBV treatment is focused on CAMs. In addition to the small molecules mentioned above, there are some other small molecules currently being studied in clinical or preclinical studies, such as EDP-514 (Feld et al., 2022), RG7907 (Gonçalves et al., 2021), ABI-H3733 (Taverniti et al., 2022), ALG-000184 (Taverniti et al., 2022), GLP-26 (Amblard et al., 2021), and SHR5133 (Li et al., 2022).

### Reverse transcriptase inhibitors

Nucleoside analogues (NAs) are the most commonly administered anti-HBV treatment in clinical settings worldwide. As small-molecule drugs, NAs can directly inhibit the activity of HBV DNA polymerases, resulting in reduced virion production (Pierra Rouviere et al., 2020). In addition, they also compete with natural nucleotide substrates to interrupt HBV DNA synthesis. There are some NAs that have been approved for CHB treatment, including lamivudine, adefovir dipivoxil, entecavir, telbivudine, and tenofovir (Leowattana and Leowattana, 2022). Long-term treatment with NAs could reduce numbers of cccDNA pools in HBV-infected hepatocytes by inhibiting nucleocapsid recycling (Leowattana and Leowattana, 2022). Nevertheless, NAs cannot suppress initial cccDNA formation in newly infected hepatocytes.

The first-generation NAs are lamivudine and adefovir dipivoxil (**Table 5**). Notably, lamivudine, which can compete with cytosine during the synthesis of viral DNA, was approved by the FDA for the treatment of CHB. However, it has been reported that lamivudine resistance occurs in approximately 70% of patients after 5 years (Jarvis and Faulds, 1999). Adefovir dipivoxil, a phosphonate acyclic NA of adenosine monophosphate, was approved in 2002. However, as with lamivudine, it was found that serious drug resistance occurred in patients receiving long-term treatment with this drug (Salpini et al., 2013).

**Table 5:** The summary of NAs.

**Structure**	**Company**	**Activity**	**PK/PD**	**Clinical status**
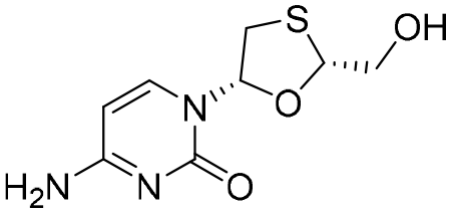 **Lamivudine**	GSK	Lamivudine inhibited DHBV replication with an IC_50_ of 0.55 μM	In human: *F*% = 86%; *C*_max_ (μg/mL): 1.5 ± 0.5; Vd (L/kg): 1.3 ± 0.4; PPB: < 30%; CL (mL/min): 199.7 ± 56.9	Approved
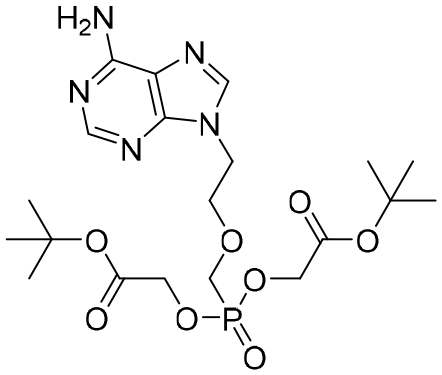 **Adefovir dipivoxil**	Gilead Sciences Inc.	In HBV transfected human hepatoma cell lines, IC_50_: 0.2–2.5 μM	In human: *F*%: 59%; *C*_max_ (ng/mL): 18.4 ± 6.26; *T*_max_ (h): 0.58–4; AUC (h × ng/mL): 220 ± 70.0	Approved
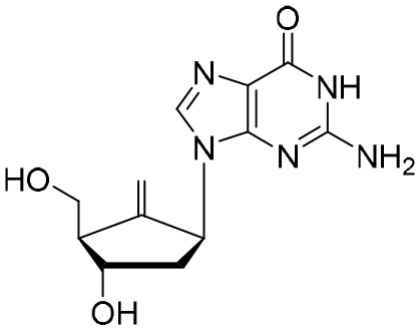 **Entecavir**	BMS	Entecavir inhibited HBV replication with an EC_50_ of 4 nM	In human: *F*%: 100%; PPB%: 13%; CL (mL/min): 383.2 ± 101.8	Approved
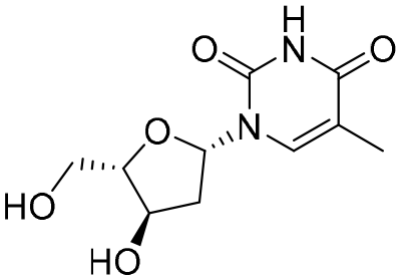 **Telbivudine**	NVS	Telbivudine inhibited anti-compliment or second-strand DNA	PPB%: 13% *in vitro* study; CL (L/h): 7.6 ± 2.9	Approved
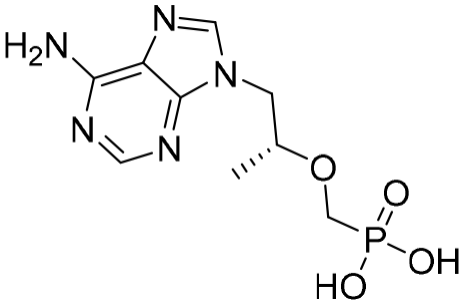 **Tenofovir**	Gilead Sciences Inc.	Tenofovir inhibited HBV replication	In human: low bioavailability; *C*_max_ (I.V.): 2,500 ng/mL; PPB%: 7.2%; Vd (L/kg): 0.813; AUC (h × ng/mL): 4,800	Approved
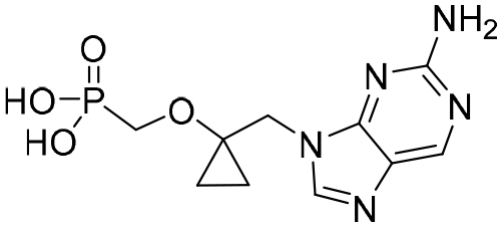 **Besifovir**	Ildong Pharmaceutical	Besifovir inhibited HBV replication and the activity was similar to that of entecavir	Undisclosed	Approved
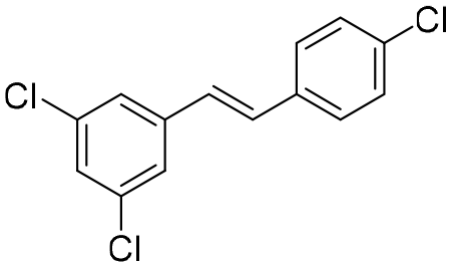 **PDM2**	NIID (Japan)	PDM2 inhibited HBV replication with an IC_50_ of 14.4 ± 7.7 μM	Undisclosed	Not available

HBV, hepatitis B virus; NAs, nucleoside analogues. Clearance (CL), Area Under Curve (AUC), Per Os (PO), Pharmacokinetics (PK), Pharmacodynamics (PD), concentration for 50% of maximal effect (EC50), 50% cytotoxicity concentrations (CC50), t1/2: half-life; Cmax, maximum concentration; Tmax, time to peak.

Entecavir, telbivudine, and tenofovir are second-generation NAs with a high genetic barrier to HBV resistance. In 2005, entecavir, a selective NA against HBV, was marketed, and its EC_50_ reached 4 nM, which was much more potent than that of lamivudine and adefovir dipivoxil (Shepherd et al., 2009). Telbivudine, an acyclic NA with activity against retroviruses, was approved in 2008 (Magalhães-Costa et al., 2015). Although telbivudine resistance was relatively low, it was associated with renal toxicity and Fanconi syndrome. Recently, tenofovir, which is suitable for the treatment of CHB patients at risk of renal dysfunction, was approved as an alternative to telbivudine because it induces fewer side effects (Childs-Kean et al., 2018). In addition, besifovir, a derivative of tenofovir, was approved by the Korean Ministry of Food and Drug Safety in 2017 for CHB treatment (Song and Park, 2021), and its antiviral efficacy was found to be similar to that of entecavir. However, the side effect of L-carnitine depletion occurred in 94.1% of patients administered besifovir (Lai et al., 2014). A phase II clinical trial on another new NA, CMX157, was also recently conducted (Painter et al., 2007; Tao et al., 2020).

Except for the inhibitors acting on the active site of HBV reverse transcriptase enzymes, the compounds could bind with the allosteric pocket being reported. PDM2, a stilbene derivative acquired by applying the high-throughput screening method, was able to inhibit HBV replication with an IC_50_ of 14.4 ± 7.7 μM, and inhibited the replication of HBV, rather than blocking its entry (Nakajima et al., 2020). In the meantime, the surface plasmon resonance (SPR) analysis demonstrated a specific interaction between PDM2 and HBV reverse transcriptase enzymes. Importantly, PDM2 showed similar inhibitory activity against the replication of both wild-type HBV and lamivudine/entecavir-resistant HBV variants (Nakajima et al., 2020).

### Ribonuclease H inhibitors

The HBV reverse transcription process is catalyzed by reverse transcriptase and ribonuclease H (RNaseH) (Shih et al., 2018). Inhibition of the activity of RNaseH causes the synthesis of strand DNA to be truncated, which in turn suppresses the formation of cccDNA (Tavis et al., 2019). Thus, RNaseH, a specific functional domain of HBV polymerase, has become a new drug target, but development is still in the primary stages.

**Figure 5:**
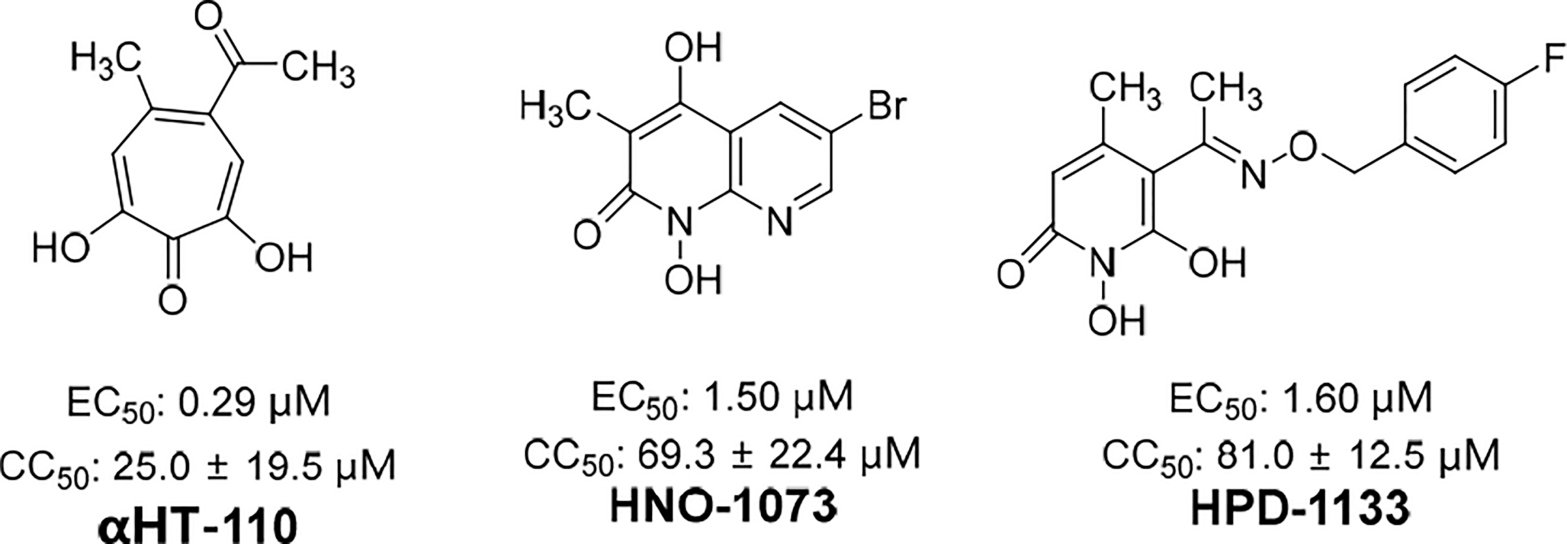
The structures of *α*-HT-110, HND-1073, and HPD-1133.

Significantly, some compounds with a unique skeletal structures were discovered to show inhibitory activity of RNaseH. Recently, three RNaseH inhibitors with different moieties, that is, *α*-HT-110, HND-1073, and HPD-1133 (**Figure 5**), that could suppress HBV replication, were identified in a study (Chauhan et al., 2021). In HBV-infected HepG2-NTCP cells, the EC_50_ values of these three compounds were between 0.29 and 1.60 μM. Comparably, the CC_50_ values were between 16 and 100 mM in HepG2-derived cell lines, indicating the non-toxicity of these compounds in primary human hepatocytes. Notably, these compounds also exhibited behaviour similar to that of lamivudine/adefovir-resistant cell lines.

### HBsAg release inhibitors

Except for complete particles, an excess of subviral particles (SVPs) were also observed. It has been found that SVPs can transport most circulating HBsAgs to [Location]: a post-ER, pre-Golgi compartment, where they interfere with innate and adaptive immunity, and therefore contribute to viral persistence (Ho et al., 2020) is a promising therapeutic method for the treatment of HDV (**Table 6**).

**Table 6:** The summary of molecules inhibiting the release of HBsAgs.

Structure	Type	Company	Activity	NCT number	Clinical status
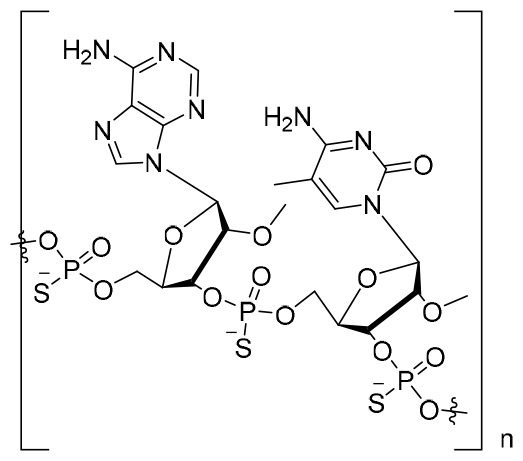 **REP 2139 (*n* = 20)**	NAP	Replicor Inc.	Both HBV and HDV infection EC_50_: 5–10 μM	NCT02233075 NCT02565719 NCT02726789	Phase II trial(s)
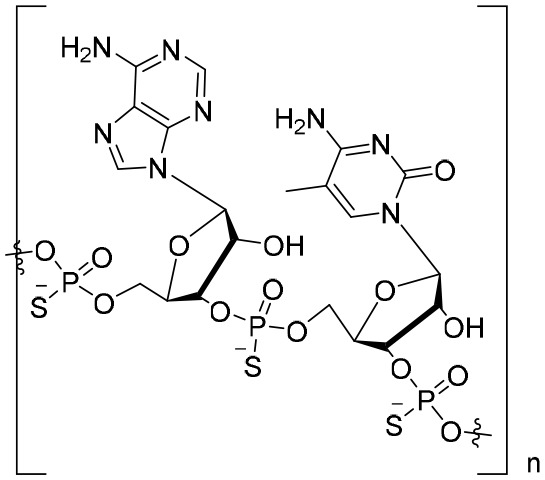 **REP 2165 (*n* = 20)**	NAP	Replicor Inc.	EC_50_: 6 μM.	NCT02565719	Phase II trial(s)
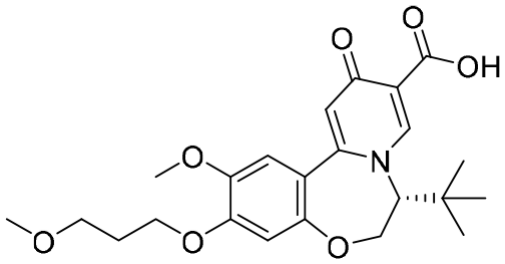 **GST-HG131**	Small molecule	Fujian Cosunter Pharmaceutical Co., Ltd	HBV-DNA EC_50_: 2.6 nM; HBsAg EC_50_: 3.9 nM	NCT04499443	Phase I trial(s)
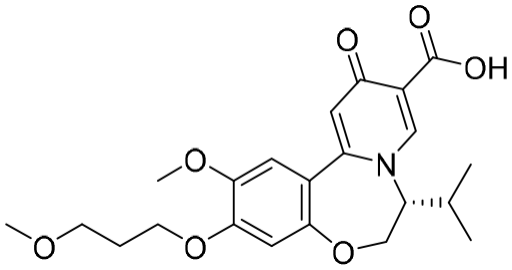 **RG7834**	Small molecule	Roche	HBV-DNA EC_50_: 0.8 nM; HBsAg EC_50_: 1.1 nM	NCT02604355	Terminated

HBV, hepatitis B virus; HBsAg, hepatitis B surface antigen; HDV, hepatitis D virus; NCT, National Clinical Trial.

Notably, nucleic acid polymers (NAPs) can disrupt apolipoprotein interactions, which are involved in the assembly and secretion of SVPs (Real et al., 2017). Recently, it has also been reported that REP 2139 and REP 2165 can reduce levels of HBsAgs in the majority of CHB patients (Vaillant, 2019). Importantly, 14 out of 40 patients achieved a functional cure status with durable HBsAg seroconversion in a clinical study (Bazinet et al., 2020).

RG7834, an orally bioavailable small molecule that belongs to the dihydroquinolizinone (DHQ) chemical class, can selectively reduce the expression levels of HBsAgs and HBeAgs *in vitro* and *in vivo* (Han et al., 2018). However, it has been announced that, because of its safety profile, the development of RG7834 will be stopped. Based on RG7834, Hu’s group designed and synthesized a series of dihydrobenzopyridooxazepine (DBP) derivatives, and GST-HG131 was discovered to be an appropriate clinical candidate (Hu et al., 2022). Notably, GST-HG131 exhibited an acceptable safety profile in healthy subjects at single doses ranging from 10 to 300 mg, and multiple doses (BID) ranging from 30 to 60 mg. Meanwhile, a multiple ascending dose study (on 30- and 60-mg doses) indicates that GST-HG131 meets therapeutic area under the curve (AUC) requirements. These corresponding experimental results indicate that GST-HG131 is a potential therapeutic option for CHB patients. In addition, the HBsAg release inhibitors in development also had LP-128, which was in a phase I trial (NCT05130567). This compound was shown to obviously reduce levels of HBsAgs, but the corresponding structure and experimental data were undisclosed.

## The treatment strategies for modulating host immunity

The discussion above focused mainly on the HBV life cycle. What follows concerns treatment strategies for host immunity. A functional cure for HBV can be achieved by appropriately orchestrating the activation of antiviral immunity (Bertoletti and Le Bert, 2018). Indeed, HBV infection can be controlled and characterized by the coordinated activation of anti-HBV-specific humoral and cellular immunity might be an effective treatment strategy (**Table 7**).

**Table 7:** The summary of compounds modulating the host immune system.

Name or structure	Target	Company	NCT number	Clinical status
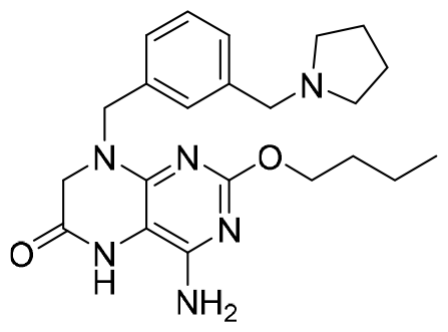 **GS-9620**	TLR7	Gilead Sciences Inc.	NCT05281510 NCT02579382 NCT04364035	Phase II trial(s)
**Undisclosed** **APR-002**	TLR7	Apros Therapeutics Inc.	NCT05268198	Phase I trial(s)
**Undisclosed** **RO7020531**	TLR7	Roche	NCT04225715	Phase II trial(s)
**Undisclosed** **JNJ-4964**	TLR7	Chia Tai-Tianqing Pharmaceutical Holdings Co., Ltd	NCT04273815 NCT04180150 NCT04202653	Phase II trial(s)
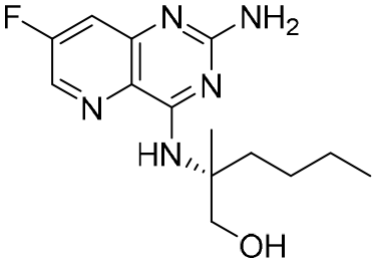 **GS-9688**	TLR8	Gilead Sciences Inc.	NCT05551273 NCT04891770	Phase II trial(s)
**iPPVO (undisclosed)** **AIC649**	TLR9	AiCuris GmBH & Co.KG	EUCTR2021–000167–69-DE	Phase II trial(s)
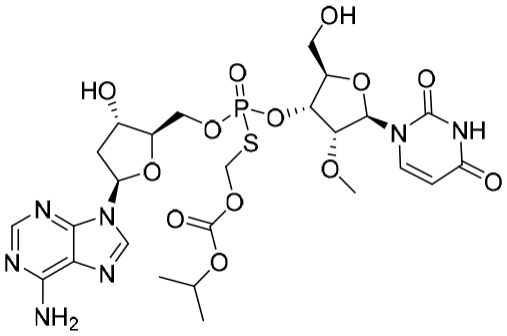 **Inarigivir soproxil**	RIG-I	Spring Bank Pharmaceuticals	NCT02751996 NCT03434353	Phase II trial(s)
**Antibody (undisclosed)** **Envafolimab**	PD-1	Ascletis Pharma Inc.	NCT04465890	Phase II trial(s)
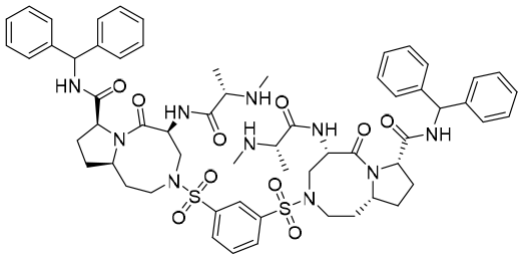 **APG-1387**	Mimicking the endogenous Smac molecule	Ascentage Pharma Inc.	NCT04568265 NCT04643405 NCT04284488	Phase II trial(s)

NCT, National Clinical Trial.

During the early phase of viral infections, the production of pro-inflammatory cytokines and IFNs, and the activation of natural killer (NK) cells are frequently observed. On account of these findings, it was shown that HBV was detected by different types of liver cells with *in vivo* and *vitro* models. Taken together with the data obtained from recent studies, this suggests that liver cell populations, as well as circulating innate immune cells, can detect and respond to HBV infection, which in turn enables the innate immune system to detect and restrict the invading virus. Therefore, it is necessary to explore the receptors and the signaling pathways responsible for detecting HBV within infected hepatocytes or other immune cells (Liu and Zhang, 2015; Naghib et al., 2022).

**Figure 6:**
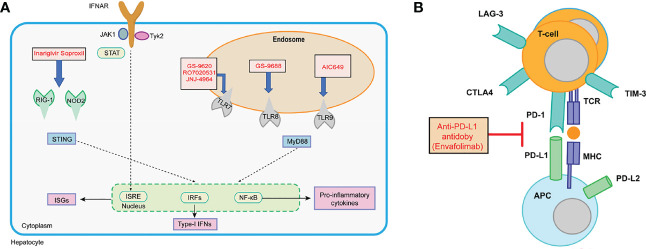
**(A)** Interferon and PRR agonism with IFN-α, RIG-I/NOD-2 agonists, TLR7 agonists, TLR8 agonist, and TLR9 agonists; **(B)** checkpoint inhibition with anti-PD-L1 antibodies.

There is a large number of new immunotherapeutic approaches in development, and some have already shown promising results (**Figure 6**). For example, it has been reported that both toll-like receptor (TLR) agonists and check-point inhibitors can restore dysfunctional HBV-specific immune responses (Gane, 2017). Similarly, vaccination is able to induce novel HBV-specific immune responses. In addition, a T-cell engineering approach has been considered as means to replace host T-cell responses (Lang et al., 2019).

### Toll-like receptor agonists

Toll-like receptor (TLR) agonists are a distinct class of pattern recognition receptors that recognize both pathogen- and damage-associated molecular patterns (Tsounis et al., 2021). TLRs induce antiviral defenses through intracellular signaling pathways that induce antiviral inflammatory cytokines and IFNs use to shape adaptive immunity (Du et al., 2022).

GS-9620, an oral agonist of TLR7, was shown to induce the prolonged suppression of viral DNA and antigens in the sera of woodchuck and chimpanzee models (Lanford et al., 2013; Menne et al., 2015). Furthermore, GS-9620 was safe and well tolerated by CHB patients. In spite of on-target biomarker responses in patients being detected, no corresponding significant declines in the levels of surface antigens were observed (Janssen et al., 2018). In attempts to optimize hepatic selectivity, APR002 was utilized, which is a novel TLR-7 agonist exhibiting a better serum-to-liver ratio than GS-9620. Importantly, APR002, in combination with entecavir, over a 12-week period was shown to cause the sustained immune-mediated suppression of cccDNA by inducing antibody production and stimulating IFN gene expression (Korolowizc et al., 2019).

RO7020531, an oral double prodrug of RO7011785, was developed for the treatment of people with CHB. At doses of 100 mg or more, RO7020531 showed acceptable safety and tolerability, and up-regulated biomarkers of TLR7 activation, including INFα- and INF-stimulated gene expression levels (Luk et al., 2020). Recently, a phase II trial for this treatment was also carried out; however, the corresponding structure and activity data *in vitro* for RO7020531 are still undisclosed.

JNJ-4964 is also an oral TLR7 agonist. In healthy adults, JNJ-4964 was well tolerated, and induced cytokines with potential anti-HBV activity rapidly, that is INFα, IP-10, IL-1 RA, MCP-1, and INF-stimulated genes (ISG15, MX1, and OAS1) in patient serum (Gane et al., 2021). In addition to RO7020531 and JNJ-4964, T7-EA, another TLR-7 agonist, displays promising pharmacodynemics (PD)/pharmacokinetics (PK) characteristics, and has been developed as a component of a therapeutic vaccine (Hu et al., 2020).

In comparison with TLR7 activation, TLR8 activation was shown to promote the production of a much higher levels of proinflammatory cystokines and chemokines, whereas it was reported that the former promoted the production of a higher levels of IFNs. (Gorden et al., 2005). GS-9688 is a TLR8 agonist in clinical development (Mackman et al., 2020), and antiviral activity has been evaluated extensively *in vitro* and *vivo* in a woodchuck model (Daffis et al., 2021). Although the therapeutic efficacy of GS-9688 in combination with NAs is still to be fully evaluated in patients with CHB, it was shown to be safe and well tolerated in a phase II trial, with a reduction in the levels of HBsAgs and HBeAgs being observed in a subset of patients (Amin et al., 2021). In addition to the trial for GS-9688, a clinical trial for its derivative, GS-9620, was carried out (Niu et al., 2018).

In addition to the TLR7 and TLR8 agonists, the TLR9 agonist was also developed for the treatment of HBV infection. AIC649 is an inactivated *Parapoxvirus ovis* (iPPVO) particle prepared with distinct immunological activities, including regulated cytokine release and the activation of T-cell responses. Currently, AIC649 is being investigated in a phase I clinical trial in patients with CHB (Paulsen et al., 2015).

### Retinoic acid-inducible gene I, nucleotide-binding oligomerization domain-like receptors agonists

The retinoic acid-inducible gene I (RIG1) and nucleotide-binding oligomerization domain-containing protein 2 (NOD2) are two types of recognition receptors that can recognize the signature patterns of foreign RNA, resulting in the activation of the IFNα signaling pathway, and the subsequent production of ISGs and proinflammatory cytokines (Sato et al., 2015). Inarigivir soproxil is a novel and oral modulator of innate immunity that is believed to activate the viral sensor proteins, that is RIG-I and NOD-2, and effects IFN-mediated antiviral immune responses in virus-infected cells (Yuen et al., 2022). The results of a phase II trial show that 12-week inarigivir soproxil, in doses of up to 200 mg, contributed to a reduction in HBV DNA, HBV RNA, and antigen levels. However, a larger reduction in the levels of HBsAgs was observed in inarigivir soproxil-pretreated patients after switching to tenofovir, although adverse events occurred in 4.7% of patients treated with the second regimen. Further studies using this new class of agents in combination with NAs to establish antiviral efficacy and safety in CHB patients are ongoing (Yuen et al., 2022).

### T-cell engineering

T cells are crucial players in the coordination of the adaptive immune response. It has been shown that CD4+ T cells contribute to the activation of B cells and regulate their differentiation into antibody-producing plasma cells (Zhu and Paul, 2010). In addition, they can also promote the formation and proliferation of memory CD8+ T cells so that they respond directly to pathogen-infected cells. CD4+ T cells can secrete a variety of cytokines and generate specific environmental stimuli that are responsible for the activation of APCs and the development of specific types of T effector cells (Zhu and Paul, 2010). However, the achievement of sustained activity, T-cell delivery, cell volume, and frequency of infusions are challenges that still need to be overcome. Recently, some relevant research has been published based on the findings of three clinical trials (i.e., NCT03899415, NCT02719782, and NCT02686372) (Bertoletti and Tan, 2020).

### Immune checkpoint inhibitors

It has been reported that programmed death receptor 1 (PD-1), the most highly expressed inhibitory receptor in HBV-specific T cells, together with the increased expression of PD-L1 (PD-1’s ligand) in HBV-infected hepatocytes, contributes to the exhaustion of T cells and therefore, high HBV replication in CHB patients (Tao et al., 2020).

Envafolimab is a single-domain antibody generated by a fusion of the PD-L1 domain with the Fc fragment of human IgG1 antibody (Markham, 2022). This chimeric molecule can bind to PD-L1 in a high-affinity manner and inhibit the PD-1/PD-L1 pathway, therefore improving T-cell function (Zhang et al., 2017a). Currently, a phase II trial evaluating the safety, tolerability, and efficacy of envafolimab in CHB patients is underway (Kim et al., 2022).

APG-1387 is a novel Smac mimetic and a highly specific antagonist of apoptosis proteins, which was independently developed by Ascentage Pharma in China (Liu et al., 2018). It degrades apoptosis proteins by mimicking the endogenous Smac molecule to induce programmed cell death or apoptosis. In a phase I trial, APG-1387 was administered intravenously at escalating dose levels (7, 12, 20, and 30 mg), followed by a 12-week observation period. Thirty patients experienced adverse events. At the end of APG-1387 treatment, a significant decline in the levels of HBV DNA, HBsAgs, and HBeAgs was observed in the cohorts administered with the 12- and 30-mg doses. In addition, APG-1387 demonstrated synergistic effect with sequential NA treatment (Rasco et al., 2019).

## Natural products

NPs have a high level of molecular complexity and diversity (except for their small molecules and RNA), and this presents a great opportunity to find novel anti-HBV drugs or lead compounds with specific antiviral mechanisms. Approximately over 160 anti-HBV NPs have been found and can be classified according to their chemical classes, that is terpenes, lignans, phenolic acids, polyphenols, lactones, alkaloids, and flavonoids (Liu et al., 2020). The following discusses the properties and mechanisms of action of representative NPs (**Table 8**).

**Table 8:** A summary of representative NPs.

Compound	Structure	Source	Mechanism of action
**Punicalin**	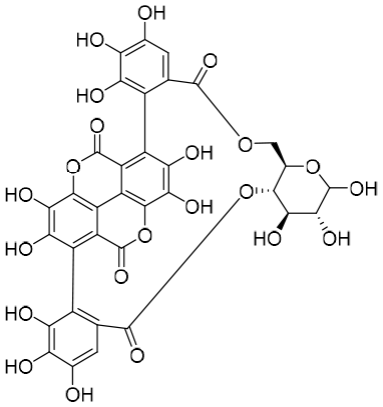	*Punica granatum* L.	It inhibits HBV cccDNA production *via* a dual mechanism, which entails the formation of cccDNA and the promotion of cccDNA decay
**Curcumin**	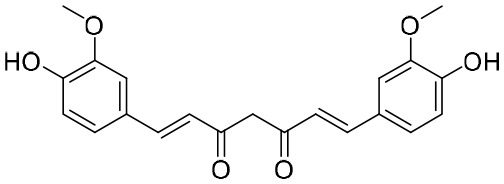	*Curcuma* L.	Down-regulating cccDNA-bound histone acetylation and metabolic co-activator PGC-1α
**Oxymatrine**	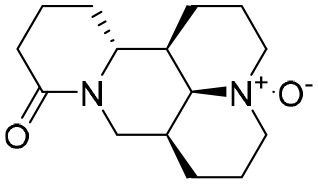	*Sophora* L.	Host Hsc70 was identified to be the target of oxymatrine
**EGCG**	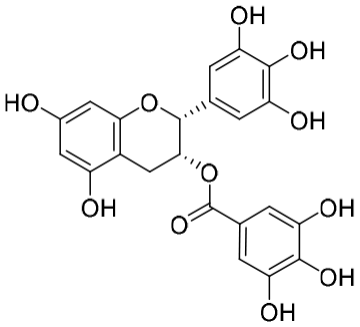	Green tea	EGCG simulates the endocytosis and degradation of NTCP by inducing the transport of NTCP from the plasma membrane to the cytoplasm
**Asiaticoside**	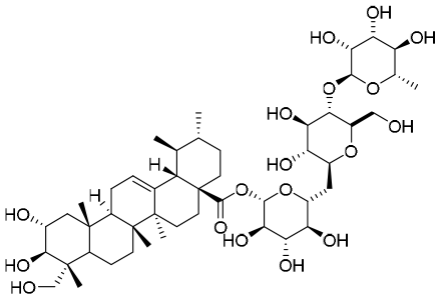	*Hydrocotyle sibthorpioides*	HBV DNA transcription and replication are affected by inhibiting the activity of C, S, and X gene promoters
**HE-145**	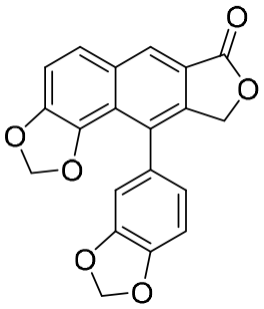	*Taiwania cryptomerioides*	HE-145 inhibits HBV DNA replication by selectively inhibiting the activity of S gene promoters II and C gene promoters in liver cancer cells
**BetA**	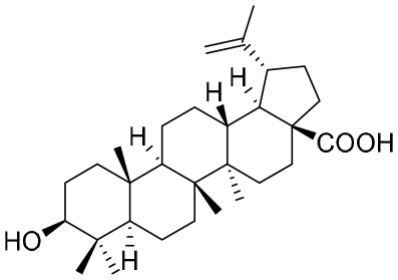	*Pulsatilla chinensis*	BetA inhibits HBV replication by down-regulating SOD2 expression
**LPRP-Et-97543**	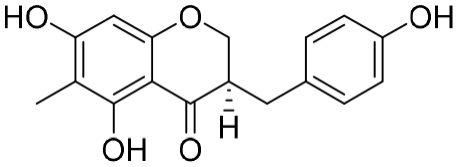	*Liriope platyphylla*	LPRP-Et-97543 inhibits HBV gene expression by interfering with the NF-κB signaling pathway in host cells

cccDNA, covalently closed circular DNA; EGCG, epigallocatechin-3-gallate; HBV, hepatitis B virus.

### Hydrolyzable tannins

By screening a compound library derived from Chinese herbal remedies, punicalagin, punicalin, and geraniin were identified as novel anti-HBV agents. Hydrolyzable tannins can inhibit HBV cccDNA production and promote cccDNA decay *via* a dual mechanism (Liu et al., 2016). Therefore, hydrolyzable tannins may serve as lead compounds for the development of new agents to cure HBV infections (Liu et al., 2016).

### Curcumin

Curcumin was isolated from the rhizome of *Curcuma longa* L., which exhibited antimicrobial activity against various bacteria, viruses, fungi, and parasites (Wei et al., 2017). Of note is that curcumin could inhibit HBV replication and expression through its reduction of cccDNA-bound histone acetylation (Wei et al., 2017). Accordingly, curcumin has the potential to be developed as a cccDNA-targeting antiviral agent for the treatment of hepatitis B. Furthermore, it has been shown that siRNAs targeting HBV act synergistically with curcumin, resulting in the latter’s enhanced inhibition of HBV infection (Wei et al., 2017).

### Oxymatrine

It has been shown that oxymatrine (OMTR), an alkaloid extracted from the Chinese herb *Sophora alopecuroides* L., has the capacity to suppress HBV (He et al., 2016). Host Hsc70 was identified to be the target of OMTR (Wang et al., 2010). OMTR (orally for 12 months) reduced blood HBV DNA and HBeAgs levels by 96% and 70%, respectively, in CHB patients resistant to lamivudine. A liver biopsy study demonstrated that OMTR caused a decrease in Hcs70 mRNA in liver cells and a reduction in intracellular HBV DNA. In combination with lamivudine (*n* = 15) (orally for12 months), OMTR also demonstrated an enhanced anti-HBV effect compared with lamivudine monotherapy (*n* = 25) (Wang et al., 2011). OMTR has been approved for the treatment of hepatitis B infections by the China Food and Drug Administration (CFDA), and was recommended as an anti-HBV agent (He et al., 2016).

### Epigallocatechin-3-gallate

It had been reported that epigallocatechin-3-gallate (EGCG), a major component of green tea, is an important transcriptional regulator of the HBV genome and can interact with Farnesoid X receptor alpha (FXRα) (Xu et al., 2016). It has also been shown that EGCG exhibits inhibitory activity against HBV infection and replication in HuS-E/2 cells (Lai et al., 2018). In addition to those discussed above, there are other NPs capable of anti-HBV activity, such as asiaticoside (Huang et al., 2013), HE-145 (Tseng et al., 2008), BetA (Yao et al., 2009), and LPRP-Et-97543 (Huang et al., 2014).

At present, a variety of NPs with novel structures and high levels of anti-HBV activity have been identified. However, the research conducted thus far is disordered and has been mainly focused on the simple isolation and identification of anti-HBV activity. More comprehensive studies about anti-HBV mechanisms and targets are relatively rare. In addition, the research carried out in most of the studies was restricted to the cellular level and, in general, there was a lack of experiments carried out with animal models. To increase the role that NPs could play in the development of anti-HBV agents, it is necessary to address these problems.

In addition to NPs, some traditional Chinese medicines (TCMs) have also demonstrated a potential ability to combat HBV infection. For example, ma-huang-tang, also known as maoto, can induce the expression of a host gene tropomyosin 2 (*TPM2*) to suppress HBV production. As the safety of ma-huang-tang has already been confirmed, it is suitable for use in the development of anti-HBV treatments (Rahman et al., 2021). *Salvia miltiorrhiza* is also a commonly used TCM and contains polyphenol (lithospermic acid). Cai’s group reported that *S. miltiorrhiza* exhibited anti-HBV activity by inhibiting HBV DNA replication in HepG2.2.15 and pHBV-transfected HepG2 cells in dose- and time-dependent manners, and by reducing the levels of HBsAgs and HBeAgs in HepG2.2.15 cells, to a certain extent. In addition, *S. miltiorrhiza* reduced HBV DNA, HBsAgs/HBeAgs, and HBcAgs levels in the serum/liver tissue of HBV-HDI C57BL/6 mice during a 3-week treatment, and suppressed the withdrawal of rebound of HBV DNA and HBsAgs levels in the mice serum (Zhu et al., 2023). It has also been reported that *Iris tectorum* Maxim. (Geng et al., 2018), *Artemisia capillaris* Thunb. (Parvez et al., 2019), and *Polygonum cuspidatum* Sieb. (Sang et al., 2017) demonstrate inhibitory activity against HBV infection.

## Conclusion and perspectives

Hepatitis B virus (HBV) infection is still a great healthcare burden worldwide. It is clear that HBV cccDNA is the viral persistence reservoir and the key obstacle to the development of a cure for CHB. The complete eradication of cccDNA could result in the development of a cure for HBV infection, enabling patients to lead a normal life after undergoing finite treatment without worrying about viral rebound. Most anti-HBV drugs on the market can effectively suppress viral replication, but, in the majority of patients, none can achieve the eradication of subviral particles and cccDNA. As we know about the HBV life cycle, a large number of direct antiviral agents with unique mechanisms of action that target specific steps of the HBV life cycle are being actively discovered, and the effects of some of these have already been evaluated in HBV patients. This extensive list of different active compounds covers nearly the entire HBV life cycle, that is entry inhibitors, viral transcription inhibitors, viral polymerase inhibitors (both RT and RNase H domains), nucleocapsid assembly modulators, and HBsAg secretion inhibitors. Furthermore, restoring or enhancing innate immunity and inducing HBV-specific adaptive immune responses can be useful in the treatment of HBV. Thus, a number of new agents restoring host immunity in HBV infection are also in development. Although the NPs are important sources of drugs, the developing of anti-HBV agents still have difficulties to overcome.

**Figure 7:**
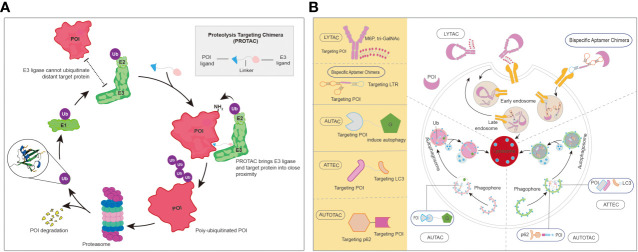
**(A)** The brief mechanism of PROTACs. **(B)** The representative of protein degradations by lysosome. AUTAC, autophagy-targeting chimera; LYTAC, lysosomal-targeting chimera; PROTAC, proteolysis-targeting chimera. AUTOTAC, Autophagy-targeting chimera; ATTEC, autophagosome-tethering compound; POI, protein of Interest.

In addition to traditional anti-HBV agents, drug designs with novel mechanisms, such as protein degradation, are also being developed. As shown in **Figure 7A**, the proteolysis-targeting chimera (PROTAC) technique was well developed, and more than 20 molecules were in clinical trails (Guenette et al., 2022). As for protein degradation by lysosomes, such as that occurring in the lysosomal-targeting chimera (LYTAC) and autophagy-targeting chimera (AUTAC) degraders, the development of this modality is still at a very early stage (**Figure 7B**) (Zhao et al., 2022). Importantly, different enzymes played significant roles during the HBV life cycle. protein degradation in the form of specialized PROTACs, could be introduced into the design of novel anti-HBV agents with the purpose of degrading the crucial enzymes or proteins involved in this specific life cycle, and might represent a promising approach to cure this disease.

## Author contributions

YP and HX wrote the manuscript. YH and SZ created the figures and edited the manuscript. ZS and WH conceived of and supervised the work. All authors contributed to the article and approved the submitted version.
